# Genome-Wide Analysis and Functional Characterization of Small Heat Shock Proteins in *Allium sativum* L. Under Multiple Abiotic Stresses

**DOI:** 10.3390/biology14101326

**Published:** 2025-09-25

**Authors:** Na Li, Bing He, Zhenyu Cao

**Affiliations:** 1 College of Horticulture and Plant Protection, Inner Mongolia Agricultural University, Hohhot 010018, China; lsf15048456237@163.com; 2 School of Business, Jiangsu Ocean University, Cangwu Road, Haizhou District, Lianyungang 222005, China; binghe@jou.edu.cn

**Keywords:** *Allium sativum*, yeast stress experiments, small heat shock proteins

## Abstract

Small heat shock proteins play a pivotal role in maintaining protein homeostasis under abiotic stress conditions and are indispensable for plant viability. In the present study, a comprehensive characterization of this gene family in *Allium sativum* was conducted through genome-wide sequence identification, phylogenetic reconstruction, conserved motif analysis, promoter cis-element profiling, transcriptomic investigation, quantitative real-time PCR, subcellular localization, and yeast-based functional assays. A total of 114 small heat shock protein genes were identified across eight chromosomes and subsequently classified into ten phylogenetic subgroups. All encoded proteins conserved the α-crystallin domain, whereas their exon–intron architectures and promoter elements responsive to environmental stress or phytohormones exhibited considerable diversity. The predicted proteins range from 130 to 364 amino acids, with isoelectric points (pI) spanning 3.97 to 9.95 and GRAVY values from −1.131 to −0.014, indicating predominantly hydrophilic characteristics. Subcellular localization analysis revealed a broad distribution across the cytoplasm, chloroplasts, mitochondria, and other compartments, with the majority (74 proteins) localized in the cytoplasm. Synteny analysis uncovered two segmentally duplicated gene pairs (*AsHSP20-80/31,* and *AsHSP20-81/32*), both showing strong purifying selection (Ka/Ks = 0.0459 and 0.2545, respectively), suggesting functional conservation. Expression profiling demonstrated predominant transcript accumulation in bulbs and floral organs, with significant induction under heat, salinity, and jasmonic acid treatments. qRT–PCR validation further confirmed that several candidate genes, notably *AsHSP20-94* and *AsHSP20-79*, were strongly and consistently upregulated across multiple stress conditions, underscoring their roles as core stress-responsive regulators. Subcellular localization experiments demonstrated that representative proteins are targeted to the cytoplasm, nucleus and chloroplasts. Furthermore, heterologous expression of *AsHSP20-79* in yeast conferred marked thermotolerance. Collectively, these findings reveal extensive expansion and functional divergence of the small heat shock protein gene family in garlic and provide valuable candidate genes for improving stress resilience in this important crop species.

## Simple Summary

Climate change and extreme weather expose garlic plants to abiotic stresses such as heat and salinity, leading to reduced yields and compromised crop quality. To elucidate the molecular mechanisms underlying garlic’s response to environmental stress, we conducted a comprehensive genome-wide analysis to identify heat shock proteins (HSPs), a class of molecular chaperones responsible for maintaining protein homeostasis by preventing misfolding and aggregation induced by stress conditions. A total of 114 *HSP* genes were identified and analyzed in terms of their subcellular localization, cis-regulatory elements responsive to stress signals, and expression patterns across various tissues and stress conditions. Functional validation in Saccharomyces cerevisiae demonstrated that overexpression of one selected *HSP* gene significantly enhances thermotolerance. These findings provide valuable insights into the molecular basis of stress adaptation in garlic and highlight candidate genes for breeding or genetic engineering aimed at improving heat and salt tolerance. Strengthening garlic’s resilience to environmental stress will contribute to stable agricultural production and food security.

## 1. Introduction

Plants are constantly exposed to various abiotic stresses, such as extreme temperatures, drought and high salinity. These environmental challenges disrupt cellular homeostasis by compromising membrane stability, altering osmotic regulation and ion balance, and leading to protein misfolding and the accumulation of reactive oxygen species (ROS) [1]. Among these, heat and salt stress are particularly harmful, as they interfere with photosynthesis, osmotic regulation and cellular metabolism, ultimately limiting growth and reducing crop yield and quality [2]. To cope with these environmental challenges, plants rely on a complex network of protective mechanisms, among which heat shock proteins (HSPs) play a pivotal role. HSPs are rapidly and robustly induced in response to abiotic stress and function as molecular chaperones that stabilize cellular proteins, facilitate proper folding, prevent aggregation and assist in the refolding or degradation of damaged proteins [3,4]. These actions are essential for maintaining proteostasis under stress conditions and ensuring cell survival. Unlike other regulatory proteins, HSPs act directly at the protein level to mitigate stress-induced damage [5,6], making them core components of the plant stress defense system. Their involvement in multiple stress-response pathways—including those triggered by heat and salinity—highlights their versatility and critical role in enhancing plant resilience.

HSPs and their regulatory networks serve as central molecular components of the plant defense system against temperature stress [7]. Eukaryotic HSPs are classified into five major families based on molecular weight: HSP110, HSP90, HSP70, HSP60 and small HSPs (sHSPs) [8]. Among these, sHSPs (also referred to as HSP20s), with molecular masses ranging from 12 to 42 kDa, contain a highly conserved α-crystallin domain (ACD) and function as the first line of defense by facilitating protein folding and preventing aggregation [9,10]. Based on sequence similarity and subcellular localization, plant sHSPs are categorized into 12 subfamilies: cytosol/nucleus CI–CVII, mitochondria MI–MII, plastid P, endoplasmic reticulum ER and peroxisome Po. Each subfamily performs specialized functions within its respective compartment, collectively contributing to acquired thermotolerance, ROS scavenging and signal regulation.

Previous studies have demonstrated that most *HSP20s* are strongly induced by various abiotic and biotic stresses—including heat, drought, salinity, cold, heavy metals, hypoxia and pathogen attack—thereby enhancing plant tolerance to these adverse conditions [11]. For example, overexpression of *MdHsp18.2b* enhances salt stress resistance [12], *Bacillus pumilus* infection resistance and anthocyanin accumulation in apple calli. In *Brachypodium distachyon*, overexpression of *BdHSP* genes improves thermotolerance [13], while *PpHSP20-32* overexpression in peach increases both plant height and heat resistance [14].

Garlic (*Allium sativum* L.) is a globally significant vegetable crop known for its abundance of bioactive constituents, including allicin, polyphenolic compounds and fructans, which collectively contribute to its pronounced antioxidant, antimicrobial and health-enhancing properties. The garlic genome is notably large, with an estimated size of approximately 16 gigabases and comprises about 91.3% repetitive sequences. Phylogenomic evidence indicates that the garlic genome has undergone three distinct whole genome duplication events. The first two duplications occurred prior to the divergence from *Asparagus officinalis*, estimated at approximately 80.8 million years ago, while the third duplication event was inferred to have occurred around 17.9 million years ago [15], and a recent transposable element burst approximately 0.2–0.3 Mya [16]. These genomic events not only contributed to the massive expansion of the garlic genome but also provided the genetic basis for the duplication, diversification and neofunctionalization of many gene families, including *HSP20s*.

In recent years, the *HSP20* gene family has been extensively characterized in various plant species such as *Arabidopsis thaliana* [17], *Zea mays* [18], *Prunus persica* L. [14] and *Cucumis sativus* [19]. These studies provide strong evidence supporting the role of *HSP20s* in enhancing plant tolerance to heat, salinity, drought and oxidative stress. Functional analyses have further demonstrated that overexpression of specific *sHSP* genes can enhance plant resilience by reducing ROS accumulation and protecting protein structures under stress conditions. Despite these advances, systematic investigations of the *HSP20* family in garlic are lacking. Prior research in garlic has primarily focused on heat shock transcription factors (HSFs) or limited members of the *HSP*70/90 families [20], with little emphasis on genome-wide identification and functional validation of the *HSP20* family. To address the current lack of systematic investigation, a total of 114 *AsHSP20* genes were identified and comprehensively characterized using the complete garlic genome sequence. A series of integrated analyses was conducted, encompassing subfamily classification, phylogenetic reconstruction, conserved motif identification, gene structure analysis, promoter cis-element profiling, tissue-specific and stress-inducible expression patterns based on transcriptome data and quantitative real-time PCR, subcellular localization and functional validation in yeast under heat stress conditions. Additional analyses included Gene Ontology enrichment and prediction of protein interaction networks. Collectively, these results bridge a critical knowledge gap in the systematic exploration of the *HSP20* gene family in garlic and offer valuable genetic resources and theoretical insights for elucidating the evolutionary mechanisms underlying small heat shock protein-mediated stress adaptation in *Allium* species, thereby facilitating the molecular breeding of stress-tolerant garlic cultivars.

## 2. Materials and Methods

### 2.1. Genome-Wide Identification of HSP20 Genes

Genomic and protein sequences of garlic were retrieved from the *Allium*DB database (https://allium.qau.edu.cn/, accessed on 18 March 2025) [21], and *Arabidopsis thaliana* sequences were obtained from the NCBI database (https://www.ncbi.nlm.nih.gov/, accessed on 18 March 2025). *A. thaliana* HSP20 protein sequences (AtHSP) were sourced from The Arabidopsis Information Resource (TAIR, version 10, http://www.arabidopsis.org, accessed on 18 March 2025) [22]. A local protein database was established, and BLASTP searches (E-value < 1 × 10^−5^) were conducted using NCBI BLAST+ (v2.11.0) to identify candidate HSP20 family members by sequence alignment. The HMM (Hidden Markov Model) profile of the HSP20 domain (PF00011) was downloaded from the Pfam database (http://pfam-legacy.xfam.org/, accessed on 18 March 2025) and applied with HMMER v3.3.2 (http://hmmer.org/, accessed on 18 March 2025) to further screen potential HSP20 proteins [23]. Candidate sequences were validated using SMART 2.8 (http://smart.embl-heidelberg.de/, accessed on 18 March 2025) [24] and the NCBI Conserved Domain Database (CDD, https://www.ncbi.nlm.nih.gov/cdd, accessed on 18 March 2025) [25] to confirm domain integrity. Physicochemical properties—sequence length, molecular weight, theoretical isoelectric point, instability index, aliphatic index and grand average of hydropathicity (GRAVY)—were computed with ProtParam (ExPASy, https://web.expasy.org/protparam/, accessed on 18 March 2025) [26]. Transmembrane regions were predicted using TMHMM 2.0 (DTU Health Tech, https://services.healthtech.dtu.dk/services/TMHMM-2.0/), subcellular localization with Cell-PLoc 2.0 (SJTU, http://www.csbio.sjtu.edu.cn/bioinf/Cell-PLoc-2/, accessed on 18 March 2025) and secondary structure with SOPMA (I-TASSER, https://npsa-prabi.ibcp.fr/cgi-bin/npsa_automat.pl?page=npsa_sopma.html, accessed on 18 March 2025).

### 2.2. Phylogenetic and Gene Structure Analyses

Full-length HSP20 protein sequences from garlic and *A. thaliana* were retrieved from UniProt (https://www.uniprot.org/, accessed on 20 March 2025). Multiple sequence alignment was performed with ClustalX 1.81 (http://www.clustal.org/clustal2/, accessed on 20 March 2025) and manually adjusted in Jalview (v2.11.2.5). A neighbor-joining phylogenetic tree was constructed in MEGA 11.0 (https://www.megasoftware.net/) using the Poisson model with 1000 bootstrap replicates. Exon–intron structures were inferred by aligning genomic and cDNA sequences and visualized using the Gene Structure Display Server (GSDS 2.0; http://gsds.gao-lab.org/, accessed on 20 March 2025). Conserved motifs were identified with MEME Suite (v5.4.1; http://meme.nbcr.net/meme/intro.html, accessed on 20 March 2025) [27], setting the maximum number of motifs to 10 and motif width between 6 and 50 residues.

### 2.3. Chromosomal Localization, Duplication and Synteny

Chromosomal coordinates of *AsHSP20* genes were extracted from the garlic genome GFF3 file and visualized using TBtools v1.09876 (https://github.com/CJ-Chen/TBtools-II, accessed on 20 March 2025) [28]. Tandem and segmental duplications were identified by MCScanX (http://chibba.pgml.uga.edu/mcscan2/, accessed on 20 March 2025) and plotted with TBtools’ “Dual Synteny Plotter” module. Interspecific synteny among garlic, onion and Welsh onion was also mapped and displayed in TBtools [28].

### 2.4. Cis-Regulatory Element Analysis, Protein–Protein Interaction Network and Gene Ontology Enrichment

Promoter regions (2000 bp upstream of the ATG) for each *AsHSP20* gene were extracted with TBtools and analyzed for cis-acting elements using PlantCARE (http://bioinformatics.psb.ugent.be/webtools/plantcare/html/, accessed on 28 March 2025) [29]. Identified elements were categorized by function—hormone response, light response, stress response and visualized as heatmaps in HemI v1.0.3.7. GO annotation analysis was conducted by extracting the DNA sequences 2000 bp upstream of the psmyb coding sequences using the GXFSequences Extract tool in TBtools. Eggnog (http://eggnog5.embl.de/, accessed on 28 March 2025) [30] was used for the GO annotation analysis, and the results were visualized using WeGo (https://wego.genomics.cn/, accessed on 28 March 2025) [31]. The protein–protein interaction (PPI) network of the AsHSP20 family was constructed using Cytoscape software (version 3.9.1). First, the protein sequences of *HSP* genes were submitted to the STRING database (https://string-db.org/, accessed on 28 March 2025) for interaction prediction. The minimum required interaction score was set to a high-confidence threshold (≥0.700) to ensure the reliability of the predicted interactions. The resulting interaction data were then imported into Cytoscape for network visualization and analysis.

### 2.5. Plant Material and Stress Treatments

The garlic variety selected is “Zipi”, and this variety is stored in the onion and garlic germplasm resource nursery of Inner Mongolia Agricultural University. Uniform, disease-free garlic cloves were surface-sterilized in 70% ethanol for 5 min, rinsed with sterile water. The plants were grown using half-strength Hoagland nutrient solution as the growth medium and germinated at 23–25 °C under a 16 h light/8 h dark cycle until seedlings reached 12–15 cm (≈3 weeks). For stress assays, seedlings were treated with 39 °C (high-temperature stress) [32], 200 mM NaCl (salt stress) [33] or 100 µM MeJA (methyl jasmonate) [34] for 6, 12 and 24 h. Control plants remained at 23 °C. Leaf samples were harvested at each time point, immediately frozen in liquid nitrogen and stored at −80 °C. For each treatment, three biological replicates were collected.

### 2.6. Quantitative Real-Time PCR

Total RNA was extracted using the RNAprep Pure Plant Kit (Tiangen Biotech, Beijing, China) and reverse-transcribed with PrimeScript™ RT Master Mix (TaKaRa Biotechnology, Dalian, China). Gene-specific primers (Supplementary Table S1) were designed with Primer 5 (Premier Biosoft, Palo Alto, CA, USA) and synthesized by Sangon Biotech (Shanghai, China). qRT–PCR was performed on an FTC-3000P Real-Time PCR System (Funglyn Biotech, Toronto, ON, Canada) using SYBR^®^ Premix Ex Taq™ II (Tli RNaseH Plus, RR820A; TaKaRa Biotechnology). GAPDH and UBQ were initially tested as candidate reference genes based on previous garlic studies, but due to their variable expression under heat (39 °C), salt (200 mM NaCl) and MeJA (100 µM) stresses, actin was ultimately selected for normalization due to its stable expression. β-Actin served as an internal control [30], and the control (CK) plants were used as an external control [35]. Relative expression levels were calculated by the 2^−ΔΔCT^ method [36]. Regarding tissue-specific expression analysis, the data were obtained from public transcriptome datasets available in the NCBI SRA database (PRJNA243415).

### 2.7. High-Temperature Stress Assay in Transgenic Yeast

The pYES2 expression vector harboring *AsHSP20-79* was constructed and introduced into Saccharomyces cerevisiae strain BY4741 using the lithium acetate/polyethylene glycol (LiAc/PEG) method. Transformed yeast colonies were selected on synthetic complete medium lacking uracil (SC–Ura) supplemented with 2% galactose to ensure selection of Ura^−^ transformants and induction of the GAL1 promoter. Yeast cells transformed with the empty pYES2 vector were included as negative controls [20]. These control strains were cultured and induced under the same conditions (SC–Ura medium supplemented with 2% galactose) and subjected to identical functional assays. Galactose-induced expression was initiated at 30 °C and 39 °C, and growth was monitored by measuring optical density at 600 nm (OD_600_) after 12 h. Cell suspensions were adjusted to an OD_600_ of 0.8, serially diluted (10^0^ to 10^−5^), and spotted onto SG-Ura plates (synthetic galactose medium lacking uracil). Plates were incubated at 30 °C, 35 °C, 37 °C, 39 °C, and 41 °C for 3 days to evaluate thermotolerance.

### 2.8. Subcellular Localization of AsHSP20 Proteins

The coding sequences of *AsHSP20* genes (excluding stop codons) were cloned into the plant expression vector *pCAMBIA1302*, which contains a CaMV 35S promoter to drive constitutive expression in plants. The resulting GFP fusion constructs and empty vector (control) were introduced into Agrobacterium tumefaciens strain GV3101 using the heat-shock method [37]. Transformed Agrobacterium cultures were grown, harvested, and resuspended in infiltration buffer (10 mM MES, 10 mM MgCl_2_, 200 μM acetosyringone, pH 5.6) to an OD_600_ of 0.8. Agroinfiltration was performed by infiltrating the bacterial suspension into the abaxial sides of fully expanded leaves from 4–6-week-old Nicotiana benthamiana plants using a needleless syringe. For co-localization analysis, Agrobacterium cultures harboring GFP constructs were mixed at a 1:1 ratio with those containing organelle-specific markers (Chlo-mCherry, NLS-mCherry or NES-mCherry). After 48–72 h of incubation under standard growth conditions, fluorescence signals of GFP and mCherry were observed using a Nikon C2 Plus confocal laser-scanning microscope (Nikon, Tokyo, Japan). Bright-field and merged images were also captured. All experiments were performed in at least three independent biological replicates.

## 3. Results

### 3.1. Identification of Family Members

To identify the *HSP20* gene family in garlic, the HSP20 protein sequences from *Arabidopsis thaliana* were used to construct a hidden Markov model (HMM). A total of *114 HSP20* genes were identified in the garlic genome and designated as *AsHSP20-1* to *AsHSP20-114* according to their chromosomal positions (Table 1). The predicted AsHSP20 proteins range from 130 (AsHSP20-27) to 364 (AsHSP20-69) amino acid residues, with calculated isoelectric points (pI) of 3.97 (AsHSP20-95)–9.95 (AsHSP20-67) and molecular weights of 15.09 (AsHSP20-25)–41.15 (AsHSP20-69) kDa. The instability indices vary between 31.01 (AsHSP20-69) and 57.43 (AsHSP20-35), while the aliphatic indices range from 54.94 (AsHSP20-95) to 102.39 (AsHSP20-78). The grand average of hydropathicity (GRAVY) values are between –1.131(AsHSP20-29) and –0.014 (AsHSP20-18), indicating hydrophilic properties. Subcellular localization predictions suggest that AsHSP20 proteins are distributed among the cytoplasm, chloroplast, nucleus, mitochondria, peroxisome, and extracellular compartments, with the majority—74 members—predicted to localize in the cytoplasm.

### 3.2. Phylogenetic Analysis

**Figure 1:**
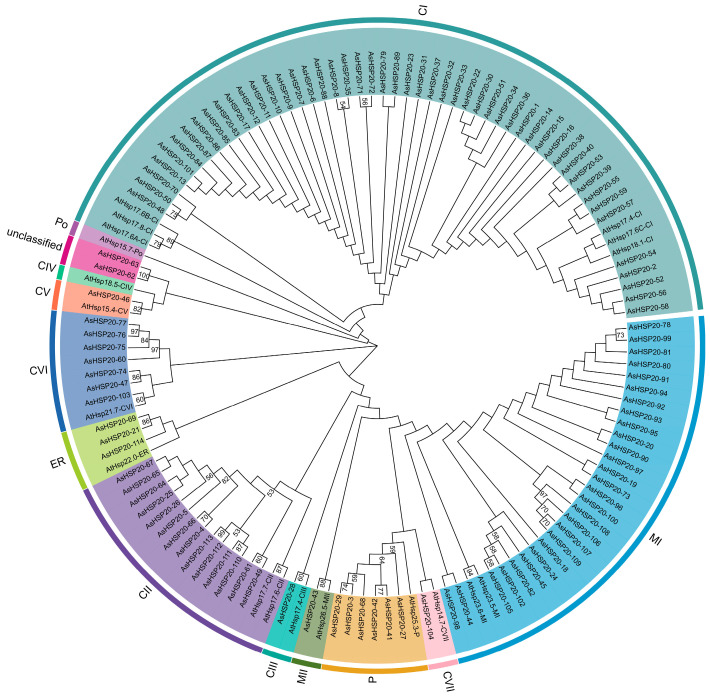
Phylogenetic analysis of the *HSP20* gene family in *A. sativum*. An unrooted neighbor-joining tree was constructed in MEGA X using the full-length amino acid sequences of 114 AsHSP20 proteins, the Poisson substitution model and pairwise deletion of gaps. Bootstrap values from 1000 replicates (shown at nodes) ≥ 50% are indicated. Based on sequence homology, the AsHSP20 proteins cluster into 10 subfamilies (CI–CIII, CV–CVII, MI, MII, ER, P) plus an unclassified group, each marked by a distinct colored arc. The scale bar represents 0.1 amino acid substitutions per site.

A phylogenetic tree was constructed based on the amino acid sequences of 19 *A. thaliana* and 114 garlic HSP20 proteins (Figure 1). According to phylogenetic clustering and predicted subcellular localization, the 114 AsHSP20 proteins were assigned to 10 subfamilies: cytosolic CI–CVII (containing 50, 14, 1, 0, 1,7, and 1 members, respectively), mitochondrial MI–MII (28 and 1), endoplasmic reticulum (ER; 3), and plastid (P; 6). Two proteins (AsHSP20*-62* and AsHSP20*-63*) did not cluster into any established subfamily and were designated as unclassified. Among the defined subfamilies, ten—CI, CII, CIII, CV, CVI, CVII, MI, MII, ER, Po (peroxisome), and P—harbor garlic HSP20 members. Notably, 74 (64.91%) of the AsHSP20 proteins belong to the cytosolic subfamilies CI–CVII, indicating that the cytosol is likely the primary functional compartment for the HSP20 family in garlic.

### 3.3. Conserved Motif and Gene Structure Analysis

**Figure 2:**
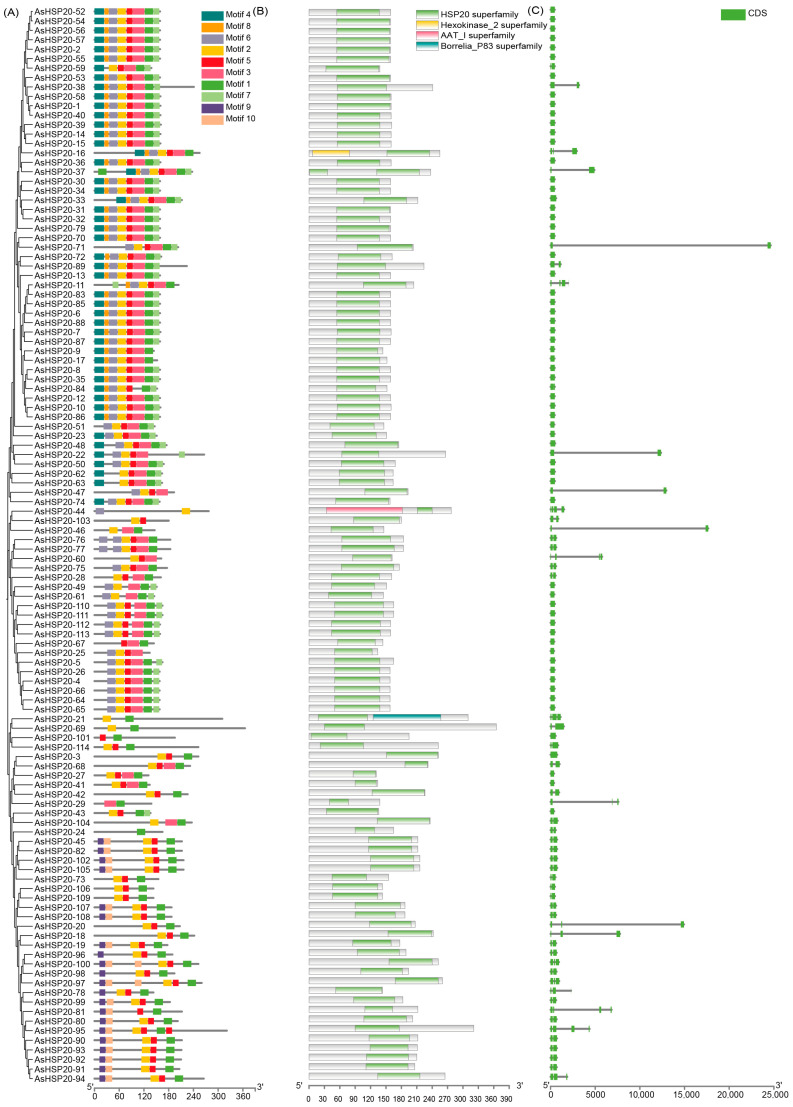
Conserved motif composition, domain architecture and gene structure of the *AsHSP20* family. *AsHSP20* genes are ordered according to their phylogenetic relationships in Figure 1. (**A**) Distribution of ten conserved motifs in AsHSP20 proteins as identified by MEME. Each colored box (motifs 1–10) corresponds to a unique amino acid signature; lengths of proteins are depicted to scale (scale bar at bottom, in amino acids). (**B**) Domain composition of AsHSP20 proteins based on Pfam annotation. Green boxes denote the HSP20 α-crystallin domain; other colors indicate ancillary domains (Hexokinase_2, AAT_I, Borrelia_P83). Unannotated regions are shown as light gray bars. Protein lengths are shown to scale. (**C**) Exon–intron organization of *AsHSP20* genes. Solid green boxes represent coding sequences (CDS), and thin black lines indicate introns; 5′ and 3′untranslated regions were omitted for clarity. Gene lengths (including introns) are drawn to scale (scale at bottom, in base pairs).

Ten conserved motifs (Motif 1–10) were identified in the AsHSP20 family using MEME (Figure 2A), with motif lengths ranging from 11 to 29 amino acids. Among these, Motif 3 is the longest (29 aa), Motif 8 is the shortest (11 aa), and Motifs 1, 2, and 6 are each 21 aa in length (Supplementary Table S2). Each AsHSP20 protein contains between 1 and 7 conserved motifs, with most members harboring 2–7 motifs; only AsHSP20-24 contains a single motif.

Most AsHSP20 proteins retain the characteristic α-crystallin domain (100–150 aa), which is essential for chaperone activity. Additionally, several members harbor extra superfamily domains, such as Hexokinase_2 (e.g., AsHSP20-47 and AsHSP20-62), AAT_I (AsHSP20-38), and Borrelia_P83 (AsHSP20-74), suggesting that these proteins may have acquired novel or extended functions through domain fusion events (Figure 2B). Gene structure analysis (Figure 2C) showed that among the 114 *AsHSP20* genes, 62 (54.4%) contain a single exon, 38 (33.3%) have two exons, 10 (8.8%) have three exons, and 4 (3.5%) possess four exons, indicating an overall simple gene architecture.

### 3.4. Chromosomal Localization

**Figure 3:**
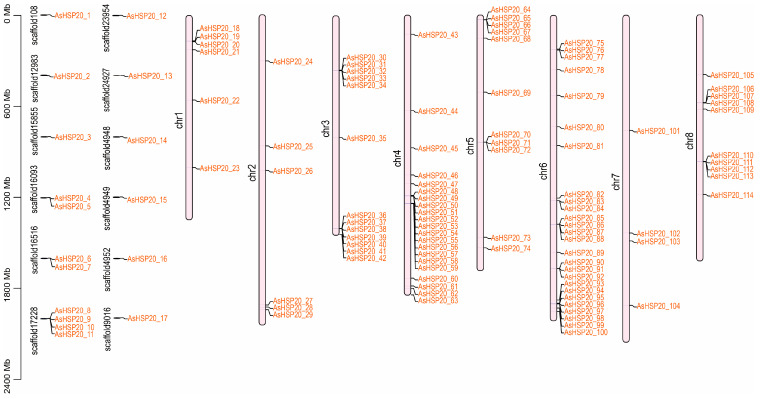
Chromosomal distribution of the *AsHSP20* gene family in *A. sativum*. The genomic positions of *AsHSP20* genes are mapped onto unanchored scaffolds (**left**) and the eight assembled garlic chromosomes (chr1–chr8; (**right**)). Scaffold IDs are shown adjacent to each scaffold, and individual *AsHSP20* genes are labelled at their approximate physical locations. A scale bar (in megabases) on the left indicates physical distance.

Chromosomal localization analysis revealed that 97 *AsHSP20* genes are distributed across the eight garlic chromosomes, while the remaining 17 genes are located on 12 unanchored scaffolds. The number of *AsHSP20* genes per chromosome ranges from 4 to 26 (Figure 3). Chromosome 6 harbors the largest cluster, with 26 genes (26.8% of the mapped members), whereas chromosome 7 contains the fewest, with only four genes (*AsHSP20-101*, *-102*, *-103* and *-104*; 4.12%). Most *AsHSP20* genes are situated in the distal regions of the chromosomes. The pronounced clustering of *AsHSP20* genes on chromosomes 4 and 6 likely reflects tandem and segmental duplication events that have driven the expansion and functional diversification of this gene family.

### 3.5. Inter Species Collinearity Analysis

**Figure 4:**
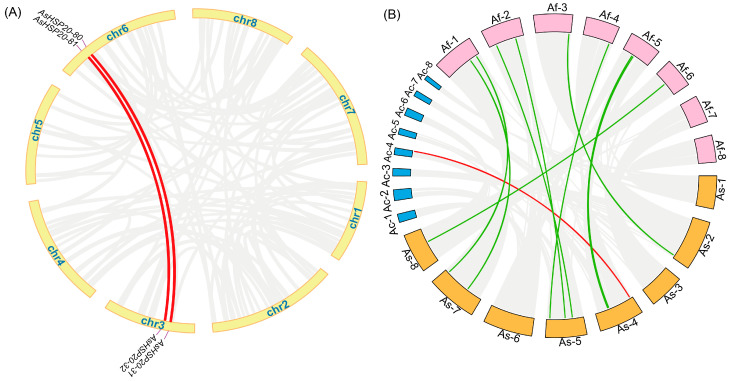
Genome wide segmental duplication and interspecies synteny of the *AsHSP20* gene family in *A. sativum*. (**A**) Circos plot depicting intraspecies segmental duplication events among *AsHSP20* genes. The outer ring represents the eight chromosomes of *A. sativum*; red ribbons link duplicated *AsHSP20* gene pairs, while gray ribbons show background genome wide collinear regions. (**B**) Synteny analysis among *A. sativum* (As, orange), *A. cepa* (Ac, blue) and *A. fistulosum* (Af, pink). Colored blocks around the circle denote individual chromosomes; ribbons indicate homologous gene pairs, with red lines marking As–Ac syntenic links and green lines marking As–Af syntenic links.

Whole genome synteny analysis of the 114 *AsHSP20* loci using MCScanX revealed two prominent segmental duplication pairs—*AsHSP20-80/AsHSP20-31* and *AsHSP20-81/AsHSP20-32* on chromosome 3 and 6—which are highlighted in (Figure 4). The Ka/Ks ratios for these pairs (0.0459 and 0.2545, respectively) are both well below 1, indicating strong purifying selection (Supplementary Table S3); the exceptionally low ratio of 0.0459 suggests an almost complete conservation at the nucleotide level, with minimal to no accumulation of non-synonymous substitutions, while the 0.2545 ratio suggests limited subfunctionalization or regulatory divergence alongside retention of core chaperone activity. To assess conservation across closely related species, *HSP20* genes from garlic (*A. sativum*), onion (*A. cepa*) and Welsh onion (*A. fistulosum*) were mapped to their respective genomes and visualized in a chord diagram (Figure 4). A single red chord marks the only one to one orthologous pair between garlic and onion (*AsHSP20-43/g513289.t1*), whereas ten green chords link garlic to Welsh onion: eight denote one to one orthologs and two (*AsHSP20-49* and *AsHSP20-52*) each correspond to two *A. fistulosum* homologs. These patterns underscore both the high conservation of the *HSP20* family within the *Allium* genus and a lineage specific expansion in Welsh onion.

### 3.6. Analysis of Cis-Acting Elements in Promoter Regions

**Figure 5:**
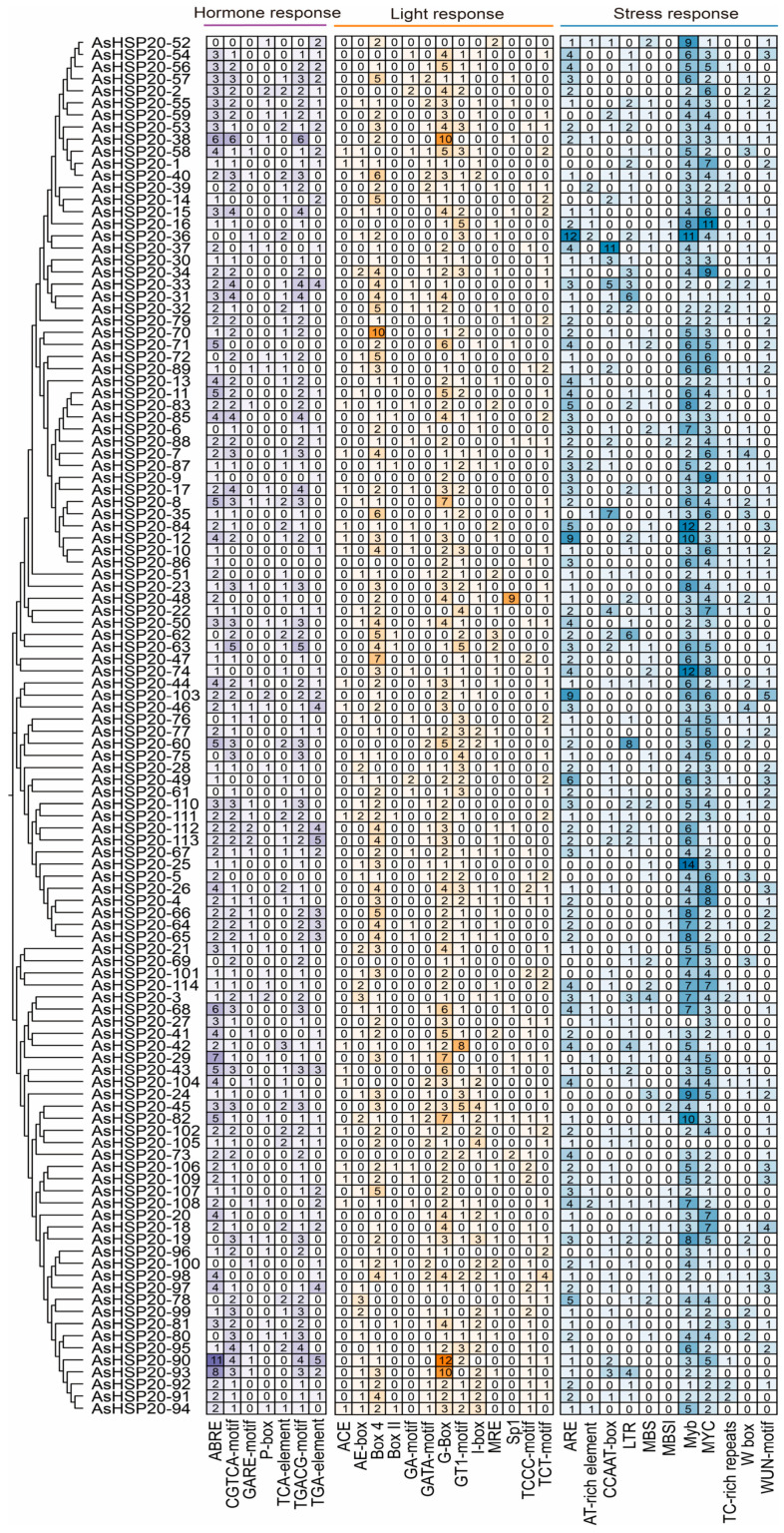
Cis-regulatory element composition in the promoter regions of *AsHSP20* genes. Promoter sequences (2 kb upstream of the translation start site) of each *AsHSP20* gene were analyzed using the PlantCARE database. The adjacent phylogenetic tree (left) clusters AsHSP20 proteins as in Figure 1. The heatmap grid shows the number of occurrences of each cis-element in individual promoters. Cis-acting elements were classified based on functional categories and represented using distinct colors: hormone responsive elements (purple), light responsive elements (yellow), and stress responsive elements (blue).

Promoter regions, defined as the 2 kilobase sequences upstream of the coding regions of all *AsHSP20* genes, were systematically analyzed for cis-acting regulatory elements and subsequently categorized into three major classes: hormone responsive, light responsive and stress responsive elements (Figure 5). Although every promoter contains a diverse array of elements, their relative abundance varies greatly, reflecting gene specific divergence in regulatory potential. Abscisic acid responsive cis-elements (ABRE) and methyl jasmonate motifs (CGTCA-motif and TGACG-motif) are ubiquitous, with particularly high densities in the promoters of *AsHSP20-90*, *-38* and *-93*, suggesting strong responsiveness to ABA and MeJA. In contrast, *AsHSP20-3* and *-8* promoters are enriched in P-box and GARE-motif sites, implicating these genes in gibberellin signaling. Core light responsive motifs such as Box 4, G-box and GT1-motif appear broadly across the family but are exceptionally abundant in *AsHSP20-90*, *-93* and *-70*, indicating potential regulation by photoperiod or light intensity. Stress-responsive elements—including low temperature response (LTR), drought response (MBS), MYC/MYB transcription factor binding sites and pathogen-related TC-rich repeats—are most prevalent in *AsHSP20-3*, *-36*, *-74* and *-16*; Hierarchical clustering of element profiles further grouped genes by regulatory complexity: for example, *AsHSP20-90*, *-93*, and *-60* share a balanced, multi-signal repertoire, whereas *AsHSP20-96*, *-27* and *-105* possess simpler element combinations indicative of more restricted control.

### 3.7. Expression Pattern Analysis

**Figure 6:**
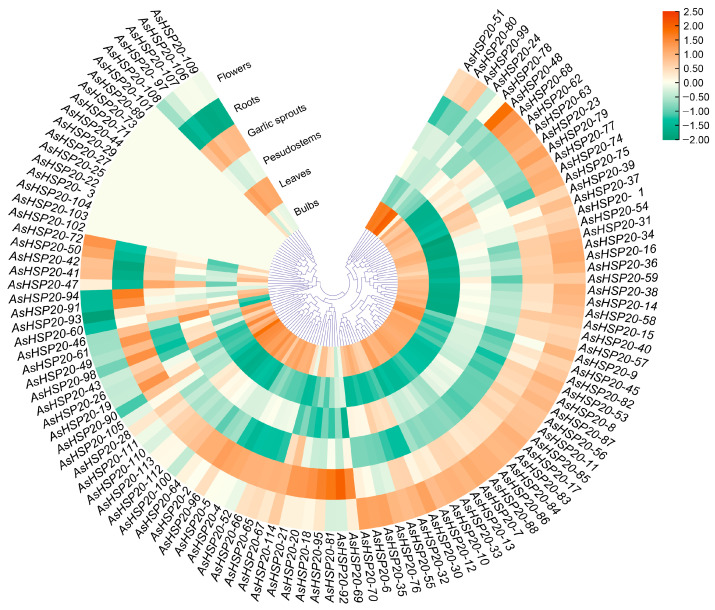
Tissue-specific expression profiles of *AsHSP20* genes in *A. sativum*. A circular heatmap illustrates the relative transcript abundance of 114 *AsHSP20* family members across six tissues: bulbs, leaves, pseudostems, garlic sprouts, roots and flowers. Genes are ordered by hierarchical clustering based on their expression patterns across these tissues (inner dendrogram), grouping members with similar profiles. Concentric rings from inside to outside represent expression in bulbs, leaves, pseudostems, garlic sprouts, roots and flowers, respectively. Transcript levels (FPKM) were log_2_-transformed and normalized; the color gradient from green through white to orange denotes low (≤−2), medium (≈0) and high (≥+2) expression.

Transcriptome profiling across six tissues (PRJNA243415) revealed distinct expression clusters among the 114 *AsHSP20* genes (Figure 6). Most members exhibit their highest transcript accumulation in bulbs and floral tissues, indicating that these organs may serve as primary sites for HSP20-mediated cellular protection. In contrast, leaves and garlic sprouts generally display low expression levels. At the individual gene level, *AsHSP20-79* demonstrates broad organ-specific expression, with particularly high transcript abundance observed in bulbs, pseudostems, sprouts and flowers; *AsHSP20-94* peaks in leaves; *AsHSP20-92* shows high expression in roots, *AsHSP20-81* was selected due to its broader organ-specific expression profile across bulbs, pseudostems, garlic sprouts, and flowers. Co-expression clustering further highlights tissue associations: *AsHSP20-79*, *-81* and *-84* cluster together with elevated expression in bulbs, roots and flowers, the majority of *sHSP* genes show markedly lower expression levels in aerial tissues such as garlic sprouts and leaves compared to bulbs, roots and floral organs. While *AsHSP20-69*, *-81*, *-91*, *-92* and *-94* are confined to underground tissues. Additionally, we identified a larger subset of genes—including *AsHSP20-3, -7, -22, -25, -27, -29, -44, -71, -73, -89, -101, -102, -103* and *-104*—that show negligible or undetectable expression under normal conditions, suggesting that they may be stringently regulated and primarily inducible under stress conditions. In contrast, genes such as *AsHSP20-26* and *-29* demonstrated preferential expression in aerial parts, particularly leaves and sprouts, implying possible functional specialization in aboveground tissue development or stress adaptation.

### 3.8. qRT–PCR Analysis

**Figure 7:**
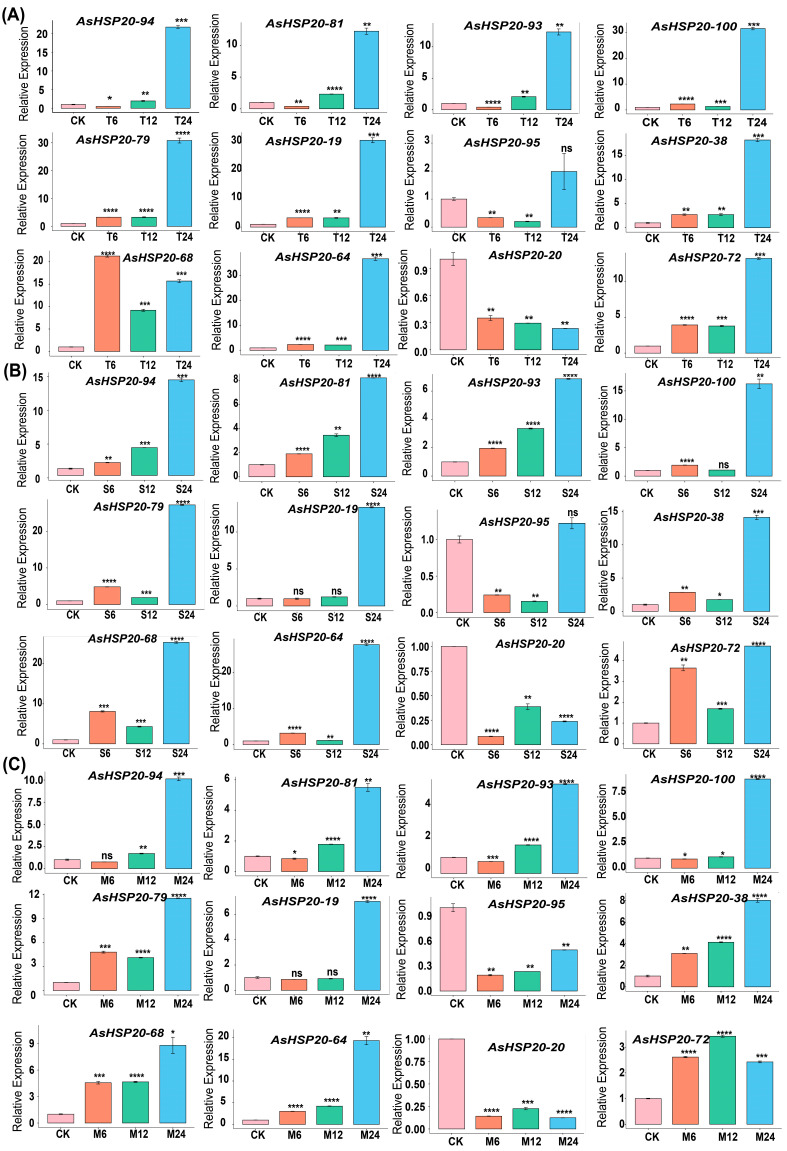
qRT-PCR analysis of selected *AsHSP20* genes in garlic under various stress treatments. Relative transcript levels of *AsHSP20s* were determined by qRT-PCR and calculated using the 2^−ΔΔCt^ method with *AsACTIN* as the internal reference. Seedlings were subjected to (**A**) heat stress (39 °C), (**B**) salt stress (200 mM NaCl) or (**C**) methyl jasmonate treatment (100 µM) for 6, 12 and 24 h; “CK”denotes untreated control plants. Data presented as mean; three biological replicates (n = 3), ±= SD of three technical replicates. Asterisks indicate significant differences relative to CK at each time point (One-way analysis of variance (ANOVA) followed by Duncan’s multiple range test: * *p* < 0.05, ** *p* < 0.01, *** *p* < 0.001,**** *p* < 0.0001; “ns” indicates not significant.

To validate the stress-responsive expression patterns of *AsHSP20* genes observed in transcriptomic analysis and clustering, we selected twelve representative members (*AsHSP20-94, -81, -93, -79, -100, -95, -64, -68, -72, -38, -20* and *-26*) for quantitative real-time PCR (qRT-PCR) (Figure 7). These genes were chosen based on their high basal expression levels in garlic leaves—the tissue most sensitive to abiotic stress and their differential responsiveness under various treatments. Leaves are typically the primary sites of physiological and phenotypic changes during environmental stress, making them an ideal tissue for identifying stress-related gene activity. Garlic plants were subjected to heat (39 °C), salt (200 mM NaCl) and methyl jasmonate (100 µM MeJA) treatments, and leaf samples were collected at 0, 6, 12 and 24 h for analysis.

Under heat stress (Figure 7A), *AsHSP20-94*, *-81*, *-93*, *-100*, *-79* and *-95* were significantly induced by 24 h, with transcript levels increasing in a time-dependent manner. In particular, *AsHSP20-79* exhibited the highest fold-change at 24 h, suggesting a central regulatory role in the heat-shock response. Conversely, *AsHSP20-38* and *-20* displayed progressive downregulation over the treatment period, indicating that these genes may be repressed by high temperature or function predominantly under non-stress conditions. Under salt treatment (Figure 7B), *AsHSP20-94*, *-81*, *-93* and *-79* again showed strong induction at 24 h, with *AsHSP20-79* reaching the greatest expression level. In contrast, *AsHSP20-95*, *-20* and *-72* exhibited negligible changes throughout the salt exposure, implying either insensitivity to salinity or involvement in more complex regulatory pathways. Following MeJA application (Figure 7C), *AsHSP20-94*, *-79* and *-64* were markedly upregulated at 24 h, with *AsHSP20-64* displaying the most pronounced response. Notably, *AsHSP20-95* and *-20* again showed sustained downregulation under MeJA treatment, while *AsHSP20-95*, and *-20* exhibited persistent repression under both salt and MeJA stresses, consistent with potential roles as negative regulators or stress-suppressed factors.

Together, these qRT–PCR results confirm that most selected *AsHSP20* genes are rapidly and robustly induced by heat, salt and MeJA, with *AsHSP20-94* and *-79* emerging as particularly versatile multi-stress markers. Meanwhile, the downregulated subset may participate in energy reallocation or negative feedback during stress responses.

### 3.9. Protein–Protein Interaction Network and Gene Ontology Enrichment

**Figure 8:**
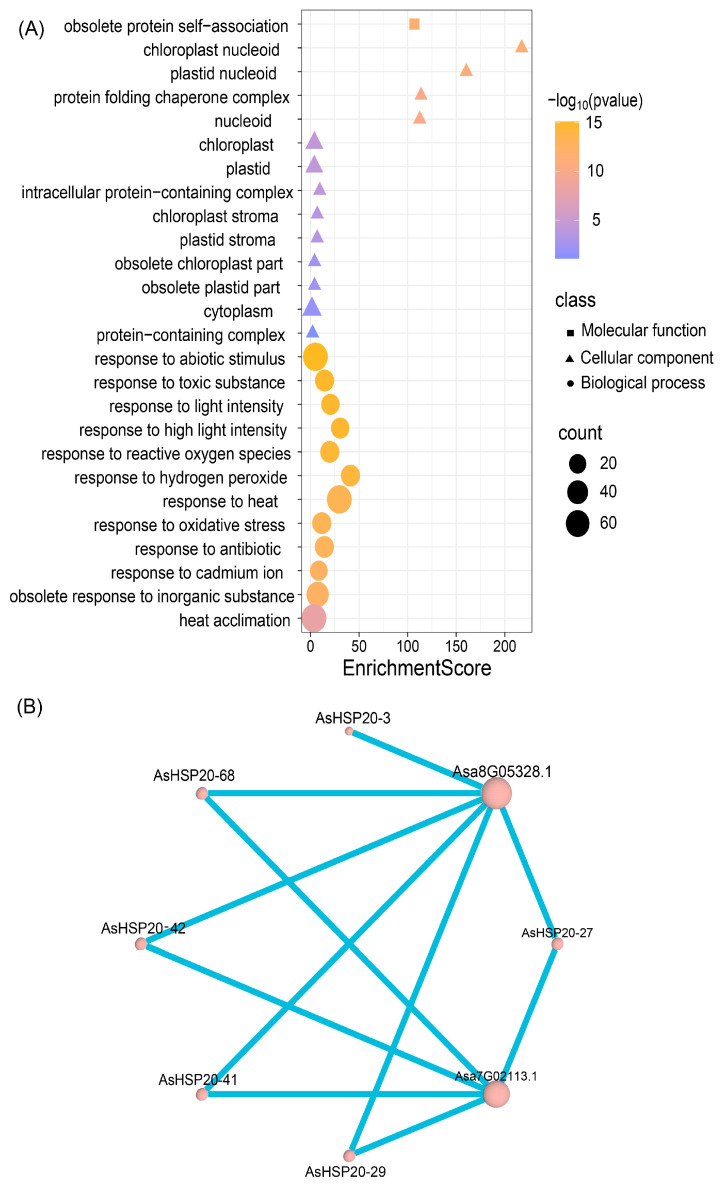
(**A**) Gene Ontology (GO) enrichment bubble plot of the heat shock protein (*HSP*) gene family in garlic. The plot displays enriched GO terms across the three main GO categories: Molecular Function, Cellular Component, and Biological Process. The x-axis represents the enrichment score, point color indicates significance level (–log_10_(*p*-value), with a gradient from blue to orange showing increasing significance), point shape denotes GO category (■ Molecular Function, ▲ Cellular Component, ● Biological Process), and point size corresponds to the number of genes enriched in each GO term. (**B**) Protein–protein interaction (PPI) network of selected *HSP* genes in garlic. Nodes represent individual *HSP* genes, with node size indicating the degree of connectivity. Edge thickness reflects the strength of interactions between genes. Asa8G05328.1 and Asa7G02113.1 are identified as central nodes with strong interactions with multiple other *HSP* genes.

To explore potential functional partnerships, Gene Ontology enrichment and predicted protein–protein interaction (PPI) analyses were performed. GO enrichment (Figure 8A) revealed significant overrepresentation of biological processes such as “response to abiotic stimulus (GO:0009628),” (Supplementary Table S4)” response to heat (GO:0009408),” “response to light intensity (GO:0009642),” “reactive oxygen species homeostasis (GO:0000304)” and “heat acclimation (GO:0010286).” At the cellular component level, terms including “protein folding chaperone complex (GO:0101031),” “chloroplast (GO:0009507)” and “plastid nucleoid (GO:0042646)” were enriched, indicating involvement in organelle-associated proteostasis. The PPI network (Figure 8B) positions several AsHSP20 proteins—namely AsHSP20-3, -42, -29 and -68—as central hubs interacting with multiple stress-related or uncharacterized partners (e.g., Asa8G05328.1, Asa7G02113.1), suggesting that *HSP20s* integrate complex signaling pathways to regulate protein stability under stress.

### 3.10. Subcellular Localization

**Figure 9:**
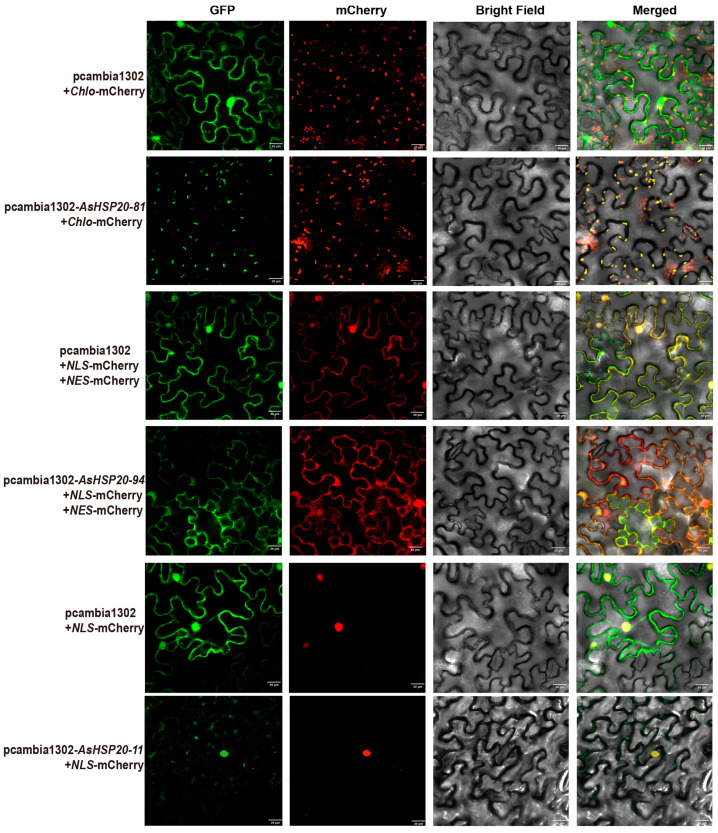
Subcellular localization of AsHSP20–GFP fusions in *Nicotiana benthamiana* leaf epidermal cells. Transiently expressed constructs were imaged for GFP fluorescence (**first column**), mCherry fluorescence (**second column**), bright-field (**third column**) and merged channels (**fourth column**). In the first row, free GFP co-expressed with the chloroplast marker Chlo-mCherry displays diffuse stromal GFP signal alongside red chloroplast fluorescence. In the second row, AsHSP20-81-GFP co-expressed with Chlo-mCherry yields a yellow merged signal within chloroplasts, indicating plastid targeting. The third row shows free GFP co-expressed with both NLS-mCherry and NES-mCherry markers, confirming dual nuclear and cytoplasmic distribution. In the fourth row, AsHSP20-94-GFP co-expressed with NLS- and NES-mCherry similarly localizes to both the nucleus and cytoplasm. The fifth row presents free GFP co-expressed with NLS-mCherry alone, demonstrating exclusive nuclear red fluorescence of the marker. Finally, in the sixth row, AsHSP20-11-GFP co-expressed with NLS-mCherry produces a yellow merged signal confined to the nucleus, revealing predominant nuclear localization of the fusion protein. Scale bars = 20 μm.

To validate the subcellular targeting of selected AsHSP20 proteins, we now clarify that AsHSP20-81, AsHSP20-94, and AsHSP20-11 were selected for subcellular localization analysis due to their distinct predicted localizations (chloroplast, cytosol, and nucleus, respectively), allowing us to assess the accuracy of the computational predictions across different compartments. GFP fusions of AsHSP20-81, AsHSP20-94 and AsHSP20-11 were transiently co-expressed in *Nicotiana benthamiana* lower epidermal cells alongside organelle markers (Figure 9): Chlo-mCherry for chloroplasts, NLS-mCherry for nuclei and NES-mCherry for cytosol. Confocal microscopy of the empty-vector controls (pCAMBIA1302 + Chlo-mCherry or pCAMBIA1302 + NLS-mCherry + NES-mCherry) confirmed marker specificity: GFP fluorescence was ubiquitous, Chlo-mCherry appeared as punctate chloroplast signals, NES-mCherry filled the cytoplasm, and NLS-mCherry accumulated in the nucleus, with no off-target overlap.

Under these conditions, AsHSP20-81-GFP produced punctate signals that overlapped almost completely with Chlo-mCherry, indicating chloroplast localization. AsHSP20-94-GFP fluorescence co-distributed with NES-mCherry but did not overlap with NLS-mCherry, demonstrating a predominantly cytosolic localization. In contrast, AsHSP20-11-GFP signals were concentrated in the nucleus, co-localizing with NLS-mCherry.

These observations reveal that AsHSP20 family members exhibit distinct subcellular partitioning—targeting chloroplasts, cytosol or nucleus—which likely underpins their diverse chaperone and regulatory functions within different cellular compartments.

### 3.11. Yeast Transgenic Verification

**Figure 10:**
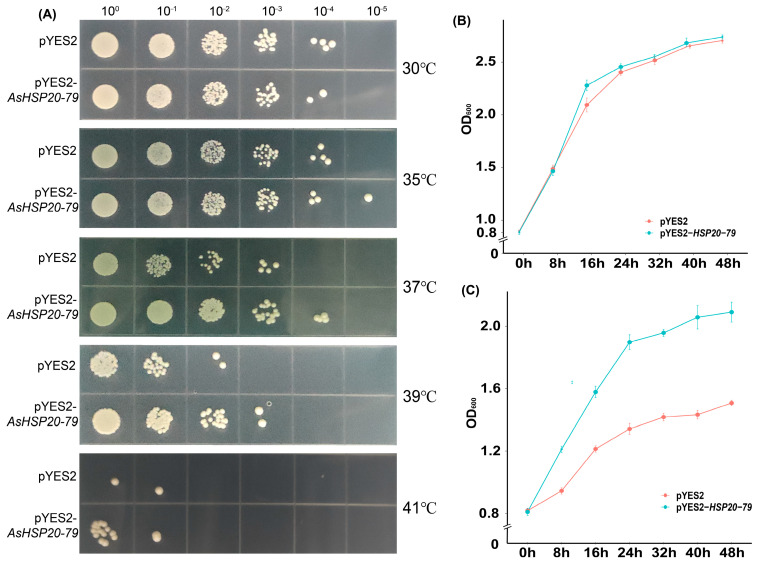
Heterologous expression of *AsHSP20-79* enhances thermotolerance in yeast. Saccharomyces cerevisiae INVSc1 cells carrying the empty vector pYES2 or the *AsHSP20-79* expression construct (*pYES2-AsHSP20-79*) were grown in SD-Ura medium to mid-log phase (OD_600_ ≈ 0.8). (**A**) Ten-fold serial dilutions (10^0^–10^−5^) were spotted onto SD-Ura agar and incubated at 30, 35, 37, 39 and 41 °C for 72 h. (**B**) Growth kinetics at 30 °C and (**C**) at 39 °C were monitored by measuring OD_600_ at 0, 8, 16, 24, 32, 40 and 48 h. Data are presented as mean ± SD of three independent experiments, demonstrating that *AsHSP20-79* expression significantly improves yeast survival and growth under elevated temperatures.

To further validate the role of *AsHSP20-79* in thermotolerance, we conducted a thermotolerance assay using a yeast expression system, selecting this gene based on its high basal expression across tissues and strong induction under various abiotic and hormonal stresses. *AsHSP20-79* was overexpressed in Saccharomyces cerevisiae, and both spot growth and liquid culture assays were performed at different temperatures. In spot assays (Figure 10A), both the control (pYES2) and the *AsHSP20-79* transformants showed comparable growth at 30 °C. At 35 °C, a slight growth advantage became evident in the transformants, which became more pronounced at 37 °C and 39 °C, where the *AsHSP20-79*–expressing strain maintained visible colony formation even at higher dilutions, while the control strain exhibited reduced viability. At 41 °C, although both strains showed limited growth, the transformants still formed stronger colonies than the control. In liquid culture, the growth curves of both strains overlapped closely at 30 °C, with OD_600_ values rising from approximately 0.9 to 2.7 over 48 h (Figure 10B), indicating that *AsHSP20-79* expression does not affect yeast proliferation under normal conditions. However, under heat stress at 39 °C (Figure 10C), the *AsHSP20-79* transformants exhibited a markedly higher growth rate starting from 16 h, ultimately reaching an OD_600_ of ~2.05 at 48 h, compared to ~1.45 for the control. This clearly indicates that *AsHSP20-79* promotes yeast cell growth and survival under high-temperature stress. Collectively, these results demonstrate that *AsHSP20-79* enhances thermotolerance in yeast, with its protective effects becoming detectable from 35 °C and intensifying at higher temperature.

## 4. Discussion

sHSPs or HSP20s constitute the largest subclass of plant heat shock proteins, acting as frontline molecular chaperones to stabilize denatured proteins, prevent aggregation, and preserve proteostasis under abiotic stress. While previous genome-wide surveys in model plants such as *Arabidopsis thaliana* [17], rice [9,38], maize [18] and potato [39] have catalogued sHSP repertoires numbering between 19 and 48 members, the identification of 114 *AsHSP20* genes in garlic represents an unprecedented expansion. This dramatic increase likely reflects both ancient and recent duplication events that have been tolerated and retained owing to the compensatory advantages they confer to this clonally propagated crop [40,41].

Chromosomal mapping revealed that *AsHSP20* loci are non-randomly distributed, with pronounced clusters in the distal regions of chromosomes 4 and 6—areas known to be hotspots for tandem and segmental duplications. The presence of highly conserved segmental duplication pairs (e.g., *AsHSP20-80/-31* and *AsHSP20-81/-32*), together with uniformly low Ka/Ks ratios (<0.3), indicates strong purifying selection preserving core chaperone functions while permitting subtle regulatory or tissue-specific divergence. Tandem duplications—evidenced by adjacent gene arrays—likely arose through unequal crossing-over or transposon-mediated recombination, further amplifying the family and potentially facilitating rapid local adaptation to fluctuating environments. In Rhododendron, most *HSP20* genes also exhibit Ka/Ks < 1 (purifying selection), yet a small subset in low-altitude species shows Ka/Ks > 1, implying episodes of positive selection linked to higher temperature or drought adaptation [42]. By contrast, all duplicated *AsHSP20* gene pairs have Ka/Ks < 0.3, demonstrating that purifying selection has uniformly dominated their evolution. To test whether any *AsHSP20* members have since acquired adaptive functions, future work should compare their expression profiles across ecological gradients (e.g., differing altitudes or abiotic stress regimes) via transcriptomic analyses.

The extreme redundancy of *HSP20* genes in garlic contrasts sharply with the smaller complements in sexual species, suggesting that a high copy number may compensate for reduced allelic diversity inherent in asexual reproduction. Indeed, gene dosage effects could amplify protective capacity under acute stress, while paralog-specific expression patterns might fine-tune responses across tissues and developmental stages. The retention of so many paralogs also raises intriguing questions about neofunctionalization versus subfunctionalization. Our discovery of extra domains, such as Hexokinase_2 [43] and AAT_I [44], in a subset of AsHSP20 proteins argues for neofunctional roles in carbohydrate metabolism or amino acid transport—functions not typically associated with classical sHSPs. It will be essential in future studies to validate these potential moonlighting activities experimentally. Analysis of *AsHSP20* gene structures uncovers two distinct patterns. Most stress-inducible paralogs are intron-poor or entirely intronless—mirroring rice, where 74% of *HSP20* genes lack introns [9], a feature likely enabling ultra-rapid transcriptional induction under heat stress [45]. By contrast, *AsHSP20s* with multiple introns may undergo alternative splicing to generate diverse isoforms, supporting tissue-specific regulation.

Subcellular localization patterns underscore the versatility of *AsHSP20* functions. Nearly two-thirds of AsHSP20 proteins localize to the cytosol, positioning them at the nexus of early stress signaling and proteome surveillance [38]. Cytosolic sHSPs likely collaborate with ATP-dependent chaperones (HSP70/HSP100) to sort misfolded substrates, decide their fate, and assist in refolding during cellular recovery [46]. Meanwhile, approximately one third of the family targets chloroplasts, organelles highly sensitive to heat and light-induced damage. The co-occurrence of *AsHSP20–PTAC5* interactions in our PPI network, together with the known role of *PTAC5* in maintaining plastid transcriptional competency, suggests that chloroplast sHSPs may stabilize not only protein complexes such as PSII and RuBisCO but also ribonucleoprotein assemblies critical for photosynthetic gene expression [47]. Such dual protection could be pivotal for sustaining photosynthetic efficiency during temperature extremes.

Promoter analysis uncovered multilayered regulatory circuits in *AsHSP20* genes, integrating hormonal, heat, and environmental signals. In addition to abundant ABRE (ABA-responsive) and MeJA-responsive motifs—which tether *AsHSP20s* to abscisic acid and jasmonate pathways (central to drought and defense responses)—we also identified numerous heat shock elements (HSEs) in their promoters, consistent with the pivotal role of heat shock transcription factors (HSFs) in driving *HSP* expression under temperature stress [48]. For example, overexpression of *PeHSFA3* in poplar activates multiple *PeHSP20* genes (*PeHSP16A*, *PeHSP22C*, *PeHSP21-2*) during heat stress, enhancing thermotolerance [49], and *AcHsfA2–1* in kiwifruit strongly induces *AcHsp20-1/2/3* promoters to improve heat resilience [50]. The particularly high density of MeJA motifs in *AsHSP20-90*, *-38*, and *-93* aligns with their strong induction by methyl jasmonate and with reports that jasmonates prime HSF activity in tomato to boost heat tolerance [51]. Meanwhile, the enrichment of light-responsive elements in *AsHSP20-90*, *-93*, and *-70* suggests potential diurnal control, possibly synchronizing chaperone accumulation with daily temperature peaks.

Tissue-specific profiling of *AsHSP20s* highlights both conserved and divergent patterns when compared across species. In geophytes such as *Allium cepa* [52], small HSP transcripts accumulate at high basal levels in the bulbs even under non-stress conditions, paralleling the strong constitutive expression of *AsHSP20s* in *A. sativum* bulbs. Likewise, the elevated *AsHSP20* abundance in floral organs echoes the petal-specific induction of *sHSP17.5-CI* in rose during flower opening [53]. In photosynthetic tissues, *AsHSP20-93* shows a leaf-restricted pattern similar to *Arabidopsis thaliana sHSP17.6*, which localizes to chloroplasts and maintains proteostasis during normal growth [54]. Root-specific expression of *AsHSP20-92* likewise mirrors reports in rice, where *OsHSP17* family members protect root meristems from soil temperature fluctuations [55]. Intriguingly, a subset of *AsHSP20* genes is virtually silent under control conditions—akin to the “inducible reserve” pools described in soybean and Arabidopsis that only activate under severe stress [56]. These latent paralogs may represent untapped resources for engineering stress resilience in specific organs.

Under heat stress (39 °C), *AsHSP20-79* transcripts rose sharply and peaking at ~30-fold by 24 h—mirroring the rapid induction of *ClHSP20-7* in watermelon (up to 18-fold at 4 h) [57] and several *GhHSP20* paralogs in cotton (>10-fold at 3 h) [58]. In response to 200 mM NaCl, *AsHSP20-79* exhibited sustained upregulation, comparable to the salt-induced expression of *JrsHSP17.3* in walnut (Juglans regia; 7–9-fold) [59] and *CaHSP22.0* in pepper (6–8-fold) [60]; conversely, *AsHSP20-38* and *AsHSP20-20* were repressed by ~2–3-fold after 24 h, reflecting the downregulation of *AsHSP17* in creeping bentgrass and *MdHsp18.2b* in apple calli under prolonged salt exposure [12,61]. Following 100 µM MeJA treatment, *AsHSP20-79* and *AsHSP20-64* were broadly induced (15–2-fold), akin to the MeJA responsiveness of *SlHSP17.7* in tomato (5–10-fold) [51]. Conversely, the observed downregulation of *AsHSP20-20* under prolonged stress may be mediated by class B heat shock factors (HSFB1/HSFB2b), which in *Arabidopsis* have been shown to repress heat inducible *HSP* genes during extended stress and recovery, thereby preventing hyperactivation of the heat shock response and facilitating homeostatic restoration [62]. By contrast, the broad and sustained upregulation of *AsHSP20-79* across heat, salt, and MeJA treatments suggests integration into multiple signaling cascades—possibly mediated by crosstalk between HSFs and jasmonate-responsive transcription factors (e.g., MYC2), as well as stress-activated MAPK modules—that coordinate both abiotic stress responses and defense hormone signaling [63]. These data demonstrate that distinct *AsHSP20* members are differentially wired into heat, salt and jasmonate signaling networks, in line with patterns seen across diverse angiosperms.

GO enrichment analysis of *AsHSP20s* revealed significant overrepresentation of “response to reactive oxygen species” and “response to hydrogen peroxide,” as well as “response to light intensity”, “chloroplast “and “heat acclimation”. These enrichments align with its predicted chloroplast localization and suggest that *AsHSP20* limits ROS accumulation [8] and preserves chloroplast function by stabilizing antioxidant enzymes or safeguarding PSII reaction centers [47]. Additionally, *AsHSP20* may interact with *PTAC5* to maintain or reassemble the PEP transcription complex, ensuring continued expression of plastid-encoded genes under heat stress [64]. In contrast, wheat *TaHSPs* [65], while sharing core chaperone activities such as protein folding and ATP binding with *AsHSP20*, display a broad subcellular distribution—including cytosol, mitochondria, endoplasmic reticulum, and chloroplasts—reflecting wider functional diversity. *AsHSP20’s* specialization in chloroplast protection thus underscores its species- and organelle-specific role within the *HSP20* family.

Because stable genetic transformation of *A. sativum* is technically challenging and time-consuming, we used Saccharomyces cerevisiae as a preliminary eukaryotic system for functional screening. Overexpression of *AsHSP20-79* in yeast conferred a clear growth advantage at 37–39 °C compared with empty-vector controls, paralleling results with CI-subfamily HSP20s from low-altitude Rhododendron species, which also enhanced yeast thermotolerance when overexpressed [42]. Mechanistically, the protein’s α-crystallin domain mediates oligomerization into large complexes that rapidly sequester unfolding intermediates, thereby preventing irreversible aggregation. In cooperation with the HSP70 and HSP40 machinery, it also refolds substrate proteins to sustain proteome integrity [66]. Promoter dissection revealed multiple heat shock elements (HSEs), indicating likely HSF-driven induction in planta, and emerging evidence points to modulation of sHSP activity by ROS and Ca^2+^ signaling during stress [67]. Together, these results provide preliminary evidence that *AsHSP20-79* acts as a positive regulator of cellular heat tolerance in eukaryotes and highlight its potential for engineering thermotolerance in crops.

In conclusion, this study provides the first comprehensive genomic and functional framework for the *HSP20* family in garlic, detailing its remarkable expansion, structural innovations, diversified regulatory landscapes, and pivotal roles in stress adaptation. Future work should leverage CRISPR/Cas9-mediated mutagenesis and promoter swapping to dissect in planta functions of key paralogs, as well as employ proteomics to identify endogenous client proteins. Ultimately, these insights pave the way for molecular breeding of garlic cultivars with enhanced environmental robustness, addressing the pressing need for resilient crops in a warming world.

## 5. Conclusions

This study presents the first comprehensive survey of the *HSP20* family in garlic, identifying 114 *AsHSP20* genes and uncovering remarkable diversity in their gene structures, subcellular localizations, promoter architectures and expression patterns. Evidence from phylogenetic, motif and synteny analyses indicates that the family has undergone extensive tandem and segmental duplications, driving evolutionary expansion and functional divergence. Tissue-specific and stress-inducible expression profiling—together with qRT–PCR validation and heterologous assays of *AsHSP20-79*—demonstrates that these genes are rapidly and robustly regulated by heat, salt and methyl jasmonate treatments, and that *AsHSP20-79* in particular confers thermotolerance in yeast. Collectively, these findings illuminate compartment-specific chaperone roles and promoter-mediated regulatory mechanisms by which *HSP20s* enhance abiotic stress resilience in garlic. The gene catalog and functional insights generated here lay a solid foundation for dissecting *HSP20-*driven regulatory networks and for harnessing key family members in future molecular breeding efforts aimed at improving garlic stress tolerance.

## Figures and Tables

**Table 1:** The characteristics of the *AsHSP20* gene family.

No.	Name	Sequence ID	Number of Amino Acid	Molecular Weight	Theoretical pI	Instability Index	Aliphatic Index	Grand Average of Hydropathicity	Subcellular Localization
1	*AsHSP20-1*	Asa0G00144.1	159	18,139.53	5.58	56.31	69.25	−0.695	Cytoplasm
2	*AsHSP20-2*	Asa0G00606.1	158	18,067.53	5.84	50.66	74.62	−0.687	Cytoplasm
3	*AsHSP20-3*	Asa0G01177.1	251	28,755.7	9.53	50.46	83.9	−0.514	Chloroplast
4	*AsHSP20-4*	Asa0G01213.1	157	17,658.25	5.79	35.18	75.16	−0.597	Cytoplasm
5	*AsHSP20-5*	Asa0G01214.1	164	18,416.18	6.19	33.58	70.73	−0.699	Cytoplasm
6	*AsHSP20-6*	Asa0G01290.1	158	18,040.5	5.84	49.91	71.52	−0.732	Cytoplasm
7	*AsHSP20-7*	Asa0G01291.1	159	18,154.52	5.56	52.42	71.07	−0.751	Cytoplasm
8	*AsHSP20-8*	Asa0G01440.1	158	18,056.5	5.83	52.43	70.89	−0.748	Cytoplasm
9	*AsHSP20-9*	Asa0G01441.1	143	16,504.7	5.78	56.83	68.18	−0.685	Cytoplasm
10	*AsHSP20-10*	Asa0G01442.1	159	18,127.54	5.82	52.95	71.07	−0.736	Cytoplasm
11	*AsHSP20-11*	Asa0G01443.1	203	23,184.11	5.61	38.01	64.33	−0.897	Nucleus
12	*AsHSP20-12*	Asa0G02746.1	158	17,999.45	5.83	49.91	71.52	−0.715	Cytoplasm
13	*AsHSP20-13*	Asa0G02962.1	158	18,084.51	5.58	51.91	70.89	−0.749	Cytoplasm
14	*AsHSP20-14*	Asa0G04879.1	159	18,061.43	5.43	50.45	67.99	−0.713	Cytoplasm
15	*AsHSP20-15*	Asa0G04881.1	159	18,121.54	5.43	50.51	67.99	−0.699	Cytoplasm
16	*AsHSP20-16*	Asa0G04883.1	255	28,913.75	5.02	51.01	81.76	−0.54	Cytoplasm
17	*AsHSP20-17*	Asa0G05708.1	151	17,233.36	6.33	51.53	61.99	−0.845	Cytoplasm
18	*AsHSP20-18*	Asa1G00674.1	241	26,723.78	4.98	50.14	102.7	−0.014	Mitochondria
19	*AsHSP20-19*	Asa1G00690.1	176	19,328.19	5.8	37.6	89.83	−0.241	Chloroplast
20	*AsHSP20-20*	Asa1G00698.1	206	23,916.61	9.47	46.58	83.74	−0.582	Cytoplasm
21	*AsHSP20-21*	Asa1G00886.1	309	35,742.26	8.83	40.37	65.05	−1.144	Cytoplasm
22	*AsHSP20-22*	Asa1G02085.1	265	29,746.8	9.22	47.87	71.7	−0.528	Chloroplast
23	*AsHSP20-23*	Asa1G03670.1	151	17,129.22	5.06	49.2	66.49	−0.784	Cytoplasm
24	*AsHSP20-24*	Asa2G01049.1	164	18,497.86	5.15	49.69	73.78	−0.564	Peroxisome
25	*AsHSP20-25*	Asa2G03191.1	133	15,090.12	5.74	35.3	70.45	−0.614	Cytoplasm
26	*AsHSP20-26*	Asa2G03812.1	157	17,651.22	5.59	32.87	74.52	−0.616	Cytoplasm
27	*AsHSP20-27*	Asa2G07035.1	130	15,223.41	5.38	48.28	74.85	−0.793	Cytoplasm
28	*AsHSP20-28*	Asa2G07089.1	160	17,709.24	5.76	44.12	83.31	−0.458	Nucleus
29	*AsHSP20-29*	Asa2G07141.1	137	15,715.82	9.17	35.29	66.13	−1.131	Nucleus
30	*AsHSP20-30*	Asa3G01343.1	158	18,151.61	5.99	42.95	72.78	−0.749	Cytoplasm
31	*AsHSP20-31*	Asa3G01354.1	158	18,062.45	5.82	43.72	73.35	−0.709	Cytoplasm
32	*AsHSP20-32*	Asa3G01355.1	158	18,058.42	6.19	46.95	70.89	−0.777	Cytoplasm
33	*AsHSP20-33*	Asa3G01357.1	211	24,285.48	5.77	48.47	75.78	−0.626	Extracellular
34	*AsHSP20-34*	Asa3G01358.1	158	18,130.54	5.82	41.93	70.32	−0.779	Cytoplasm
35	*AsHSP20-35*	Asa3G02930.1	158	18,198.66	5.83	57.43	71.52	−0.78	Cytoplasm
36	*AsHSP20-36*	Asa3G05026.1	159	17,964.38	5.57	50.63	67.99	−0.673	Cytoplasm
37	*AsHSP20-37*	Asa3G05028.1	236	27,183.15	9.54	49.51	71.4	−0.795	Cytoplasm
38	*AsHSP20-38*	Asa3G05030.1	240	27,130.25	5.1	45.76	65.38	−0.873	Cytoplasm
39	*AsHSP20-39*	Asa3G05031.1	160	18,020.38	5.83	47.72	67.56	−0.703	Cytoplasm
40	*AsHSP20-40*	Asa3G05032.1	159	18,163.59	5.59	55.11	67.99	−0.734	Cytoplasm
41	*AsHSP20-41*	Asa3G05113.1	133	15,672.91	5.09	39.27	68.8	−0.793	Cytoplasm
42	*AsHSP20-42*	Asa3G05114.1	225	25,733.82	9.42	48.25	74.53	−0.644	Chloroplast
43	*AsHSP20-43*	Asa4G00429.1	135	15,283.21	5.13	43.5	84.37	−0.597	Chloroplast
44	*AsHSP20-44*	Asa4G02369.1	276	31,962.66	9.07	48.86	78.01	−0.519	Nucleus
45	*AsHSP20-45*	Asa4G03225.1	211	23,503.47	6.66	56.59	80	−0.665	Chloroplast
46	*AsHSP20-46*	Asa4G03797.1	145	16,458.88	9.9	55.27	84.69	−0.51	Cytoplasm
47	*AsHSP20-47*	Asa4G04043.1	192	21,970.19	9.11	46.21	82.71	−0.66	Cytoplasm
48	*AsHSP20-48*	Asa4G04292.1	174	19,748.48	5.6	49.47	80.57	−0.444	Cytoplasm
49	*AsHSP20-49*	Asa4G04294.1	150	16,984.37	5.55	36.96	79.2	−0.593	Cytoplasm
50	*AsHSP20-50*	Asa4G04295.1	167	18,884.46	5.21	43.34	78.14	−0.432	Cytoplasm
51	*AsHSP20-51*	Asa4G04310.1	145	16,369.66	5.82	46.6	74	−0.61	Chloroplast
52	*AsHSP20-52*	Asa4G04540.1	158	18,022.45	5.84	53.82	73.99	−0.712	Cytoplasm
53	*AsHSP20-53*	Asa4G04541.1	158	18,042.55	5.73	44.33	73.35	−0.632	Cytoplasm
54	*AsHSP20-54*	Asa4G04542.1	158	17,977.45	5.58	46.32	77.66	−0.637	Cytoplasm
55	*AsHSP20-55*	Asa4G04543.1	158	17,868.2	5.57	55.65	74.62	−0.653	Cytoplasm
56	*AsHSP20-56*	Asa4G04545.1	158	17,999.37	5.4	49.05	73.99	−0.7	Cytoplasm
57	*AsHSP20-57*	Asa4G04549.1	158	18,024.42	5.84	52.17	74.62	−0.694	Cytoplasm
58	*AsHSP20-58*	Asa4G04550.1	159	18,168.52	5.57	55.48	68.62	−0.716	Cytoplasm
59	*AsHSP20-59*	Asa4G04555.1	137	15,729.83	5.56	51.07	73.94	−0.711	Cytoplasm
60	*AsHSP20-60*	Asa4G06279.1	161	18,106.77	6.84	40.72	87.14	−0.313	Cytoplasm
61	*AsHSP20-61*	Asa4G06421.1	144	16,262.56	5.54	45.56	77.08	−0.632	Cytoplasm
62	*AsHSP20-62*	Asa4G06481.1	163	18,743.39	8.75	42.67	66.81	−0.696	Cytoplasm
63	*AsHSP20-63*	Asa4G06617.1	163	18,743.39	8.75	42.67	66.81	−0.696	Cytoplasm
64	*AsHSP20-64*	Asa5G00107.1	157	17,684.29	5.79	34.7	74.52	−0.619	Cytoplasm
65	*AsHSP20-65*	Asa5G00109.1	157	17,714.32	5.52	35.18	74.52	−0.596	Cytoplasm
66	*AsHSP20-66*	Asa5G00110.1	157	17,658.25	5.79	35.18	75.16	−0.597	Cytoplasm
67	*AsHSP20-67*	Asa5G00111.1	144	16,457.79	9.95	72.56	59.51	−1.116	Chloroplast
68	*AsHSP20-68*	Asa5G00687.1	231	26,254.38	8.7	56.53	75.06	−0.615	Chloroplast
69	*AsHSP20-69*	Asa5G01992.1	364	41,148.26	7.74	31.01	61.46	−1.069	Mitochondria
70	*AsHSP20-70*	Asa5G03263.1	158	18,139.6	6.77	44.21	70.19	−0.739	Cytoplasm
71	*AsHSP20-71*	Asa5G03264.1	202	23,326.28	6.27	52.18	62.67	−1.052	Chloroplast
72	*AsHSP20-72*	Asa5G03265.1	161	18,468.97	6.2	48.03	68.32	−0.775	Cytoplasm
73	*AsHSP20-73*	Asa5G05424.1	154	18,137.42	6.76	50.47	81.04	−0.899	Cytoplasm
74	*AsHSP20-74*	Asa5G05661.1	157	18,014.73	6.21	50.18	86.24	−0.657	Cytoplasm
75	*AsHSP20-75*	Asa6G00868.1	175	19,933.25	6.66	34.13	94.69	−0.399	Extracellular
76	*AsHSP20-76*	Asa6G00869.1	183	20,814.25	6.36	35.82	92.68	−0.374	Chloroplast
77	*AsHSP20-77*	Asa6G00885.1	183	20,809.21	6.36	37.57	94.26	−0.38	Chloroplast
78	*AsHSP20-78*	Asa6G01385.1	142	15,912.53	8.56	36.17	102.39	−0.342	Mitochondria
79	*AsHSP20-79*	Asa6G02013.1	158	18,175.6	5.84	51.36	66.58	−0.782	Cytoplasm
80	*AsHSP20-80*	Asa6G02870.1	201	22,824.07	6.77	44.82	81.59	−0.53	Chloroplast
81	*AsHSP20-81*	Asa6G03303.1	211	23,694.78	9.43	44.38	75.5	−0.55	Chloroplast
82	*AsHSP20-82*	Asa6G04489.1	211	23,446.46	6.67	56.22	79.53	−0.631	Chloroplast
83	*AsHSP20-83*	Asa6G04532.1	158	18,026.48	5.83	49.37	71.52	−0.732	Cytoplasm
84	*AsHSP20-84*	Asa6G04534.1	151	17,129.42	5.56	51.47	75.5	−0.756	Cytoplasm
85	*AsHSP20-85*	Asa6G05099.1	158	18,070.59	5.83	48.04	72.72	−0.696	Cytoplasm
86	*AsHSP20-86*	Asa6G05100.1	158	17,972.39	5.82	50.45	70.89	−0.699	Cytoplasm
87	*AsHSP20-87*	Asa6G05101.1	158	18,098.58	5.83	53.27	70.89	−0.775	Cytoplasm
88	*AsHSP20-88*	Asa6G05102.1	158	18,022.49	5.84	52.35	70.89	−0.74	Cytoplasm
89	*AsHSP20-89*	Asa6G05713.1	223	25,172.88	8.42	47.45	86.05	−0.396	Mitochondria
90	*AsHSP20-90*	Asa6G06028.1	211	23,594.81	9.32	51.03	74.45	−0.654	Chloroplast
91	*AsHSP20-91*	Asa6G06030.1	205	22,974.09	5.85	37.56	72.93	−0.497	Chloroplast
92	*AsHSP20-92*	Asa6G06033.1	209	23,406.58	6.77	51.32	77.08	−0.554	Chloroplast
93	*AsHSP20-93*	Asa6G06784.1	210	23,730.91	6.86	44.27	78.57	−0.548	Chloroplast
94	*AsHSP20-94*	Asa6G06852.1	264	29,904.88	5.74	41.81	80.61	−0.528	Cytoplasm
95	*AsHSP20-95*	Asa6G06861.1	320	35,578.35	3.97	51.8	54.94	−1.213	Chloroplast
96	*AsHSP20-96*	Asa6G06867.1	188	21,325.27	6.78	43.29	86.06	−0.621	Cytoplasm
97	*AsHSP20-97*	Asa6G06873.1	259	28,998.91	8.46	48.9	81.74	−0.464	Cytoplasm
98	*AsHSP20-98*	Asa6G06874.1	193	22,206.22	9.04	42.27	77.77	−0.791	Chloroplast
99	*AsHSP20-99*	Asa6G06974.1	182	20,251.13	7.71	44.44	92.2	−0.394	Chloroplast
100	*AsHSP20-100*	Asa6G07048.1	251	28,187.72	5.66	40.82	77.69	−0.563	Chloroplast
101	*AsHSP20-101*	Asa7G02785.1	194	21,729.86	8.98	42.33	81.44	−0.582	Cytoplasm
102	*AsHSP20-102*	Asa7G05180.1	215	23,979.15	7.77	53.94	76.23	−0.601	Chloroplast
103	*AsHSP20-103*	Asa7G05384.1	179	20,697.54	5.26	47.4	76.15	−0.578	Cytoplasm
104	*AsHSP20-104*	Asa7G06920.1	235	26,777.34	9.11	56.92	72.17	−0.769	Chloroplast
105	*AsHSP20-105*	Asa8G01375.1	215	23,965.12	6.66	59.25	75.77	−0.589	Chloroplast
106	*AsHSP20-106*	Asa8G02037.1	142	16,716.65	4.95	47.33	63.1	−1.128	Cytoplasm
107	*AsHSP20-107*	Asa8G02053.1	186	21,194.91	5.41	45.58	73.49	−0.762	Chloroplast
108	*AsHSP20-108*	Asa8G02054.1	186	21,078.92	5.66	44.21	78.17	−0.659	Chloroplast
109	*AsHSP20-109*	Asa8G02220.1	142	16,716.65	4.95	47.33	63.1	−1.128	Cytoplasm
110	*AsHSP20-110*	Asa8G03628.1	164	18,250.02	6.6	33.48	78.41	−0.538	Cytoplasm
111	*AsHSP20-111*	Asa8G03629.1	164	18,248.05	6.6	32.36	79.02	−0.522	Cytoplasm
112	*AsHSP20-112*	Asa8G03632.1	158	17,662.33	6.53	35.32	75.13	−0.63	Cytoplasm
113	*AsHSP20-113*	Asa8G03635.1	158	17,648.31	6.53	34.78	75.13	−0.63	Cytoplasm
114	*AsHSP20-114*	Asa8G04457.1	251	28,493.48	9.75	35.61	60.2	−0.941	Chloroplast

## Data Availability

Data will be made available on request.

## Supplementary Materials

The following supporting information can be downloaded at: https://www.mdpi.com/article/10.3390/biology14101326/s1, Table S1: List of the primers used in the study. Table S2: List of Conserved Motifs and Corresponding Sequences in AsHSP20s. Table S3: Ka and Ks values of highlight homologous gene pairs of *HSP20* gene family in *A. sativum*. Table S4: Gene ontology (GO) annotation analysis of *AsHSP20s.*
